# Supplementary material for: Warm/cool-tone switchable thermochromic material for smart windows by orthogonally integrating properties of pillar[6]arene and ferrocene
Source: Nat Commun. 2018 Apr 30;9:1737. doi: 10.1038/s41467-018-03827-3 (PMC5928112; doi:10.1038/s41467-018-03827-3)
Supplement: Supplementary file 1 — Supplementary Information [file 41467_2018_3827_MOESM1_ESM.pdf]

Supplementary Information

Warm/cool-tone switchable thermochromic material for smart windows by orthogonally integrating properties of pillar[6]arene and ferrocene

Sai Wang, Zuqiang Xu, Tingting Wang, Tangxin Xiao, Xiao-Yu Hu,* Ying-Zhong Shen,* and Leyong Wang*

Supplementary Methods

All reagents were commercially available and used as supplied without further purification unless otherwise stated. **EGP6**, **mPEG-Fc** (M_n = 2347 g·mol^{-1}), ferrocene modified acrylamide monomer (**FcAm)**, and dry **Fc-gel** were synthesized according to the literatures.[1-3] Nuclear Magnetic Resonance (NMR) spectra were recorded on a Bruker Advance DMX 400 spectrophotometer or a Bruker Advance DMX 500 spectrophotometer with internal standard tetramethylsilane (TMS) and solvent signals as internal references at 25 ℃. Scanning electron microscopy (SEM) was carried out on a Shimadzu SSX-550 device. UV-vis spectra were recorded on a Shimadzu UV-1780 UV-vis Spectrophotometer.

Supplementary Discussion

- **Preparation of Fc-gel**

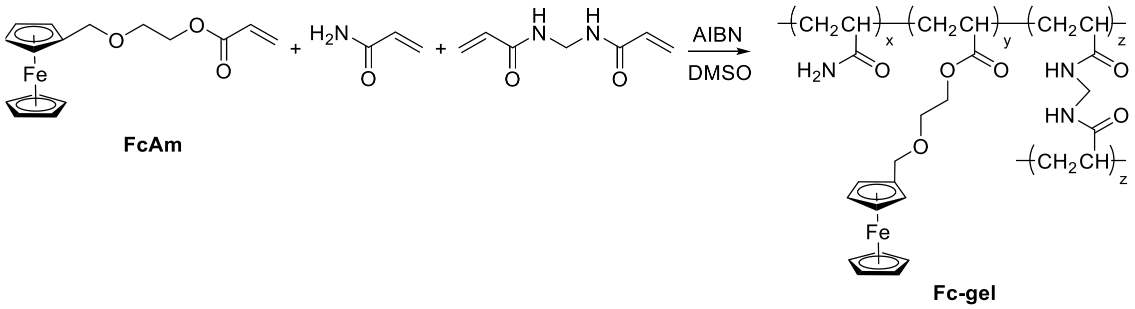

Preparation method of dry **Fc-gel**: ferrocene modified acrylamide monomer (**FcAm**) (220 mg, 0.70 mmol), acrylamide (448 mg, 6.30 mmol), and *N*,*N′*-methylenebis(acrylamide) (MBA) (5.40 mg, 0.035 mmol) were dissolved in dimethyl sulfoxide (DMSO) (1.6 mL). Then azodiisobutyronitrile (AIBN) (28.8 mg, 0.175 mmol) was added to the solution and the mixture was purged with dry argon for 30 min. The resulting solution was equally divided into 10 vials (10 mm in diameter) and sealed. The polymerization was performed in an oven at 70 ℃ for 24 h. Then, the vials were cooled down to room temperature, disc-shaped samples were washed successively by DMSO and deionized water, and subsequently dried in oven (50 ℃) under vacuum for 12 h after natural drying. Finally, disc-shaped dry **Fc-gel** was

obtained with 7 mm in diameter and 1 mm in thickness.

➢ Host–guest complexation between EGP6 and mPEG-Fc

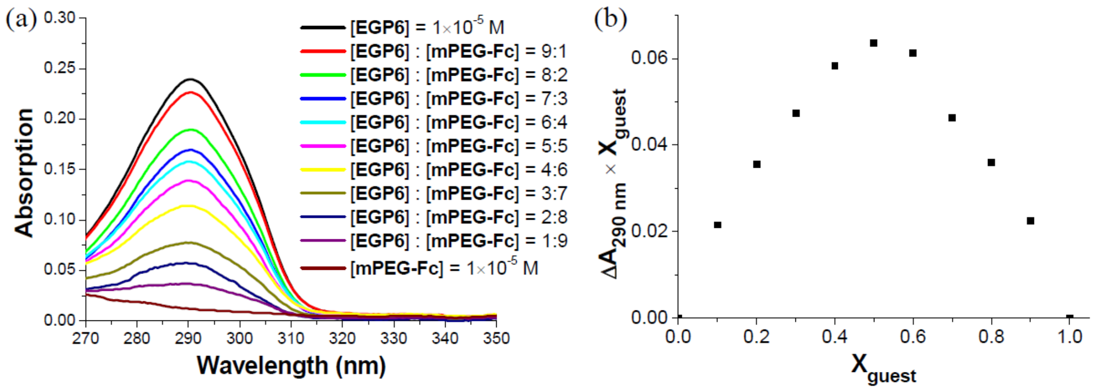

Supplementary Figure 1. (a) UV-Vis absorption of the mixture of **EGP6** and **mPEG-Fc** in water at different molar ratios while [**EGP6**] + [**mPEG-Fc**] = 1.0 × 10^{-5} M. (b) Job plot showing 1:1 stoichiometry of the complex between **EGP6** and **mPEG-Fc** by plotting the difference in absorption at 290 nm (a characteristic absorption peak of **EGP6**) against the mole fraction of guest **mPEG-Fc** with an invariant total concentration of 1.0 × 10^{-5} M in aqueous solution.

To determine the binding constant between **EGP6** and **mPEG-Fc**, ^{1}H NMR titration experiments were carried out at 298 K with a constant concentration of **EGP6** (2.0 mM) and varying concentrations of **mPEG-Fc** (Supplementary Fig. 2). The binding constant was achieved by the non-linear curve-fitting method, using the following supplementary equation 1:

$$\Delta\delta = (\Delta\delta_{\infty}/[\mathrm{H}]_0)(0.5[\mathrm{G}]_0 + 0.5([\mathrm{H}]_0 + 1/K_\mathrm{a}) - (0.5([\mathrm{G}]_0^{2} + (2[\mathrm{G}]_0(1/K_\mathrm{a} - [\mathrm{H}]_0)) + (1/K_\mathrm{a} + [\mathrm{H}]_0)^{2})^{0.5})) \qquad 1$$

Where $\Delta\delta$ is the chemical shift change of H_A on **EGP6**, $\Delta\delta_{\infty}$ is the chemical shift change of H_A when the host is completely complexed, $[H]_0$ is the initial concentration of **EGP6**, and $[G]_0$ is the varying concentrations of **mPEG-Fc**.

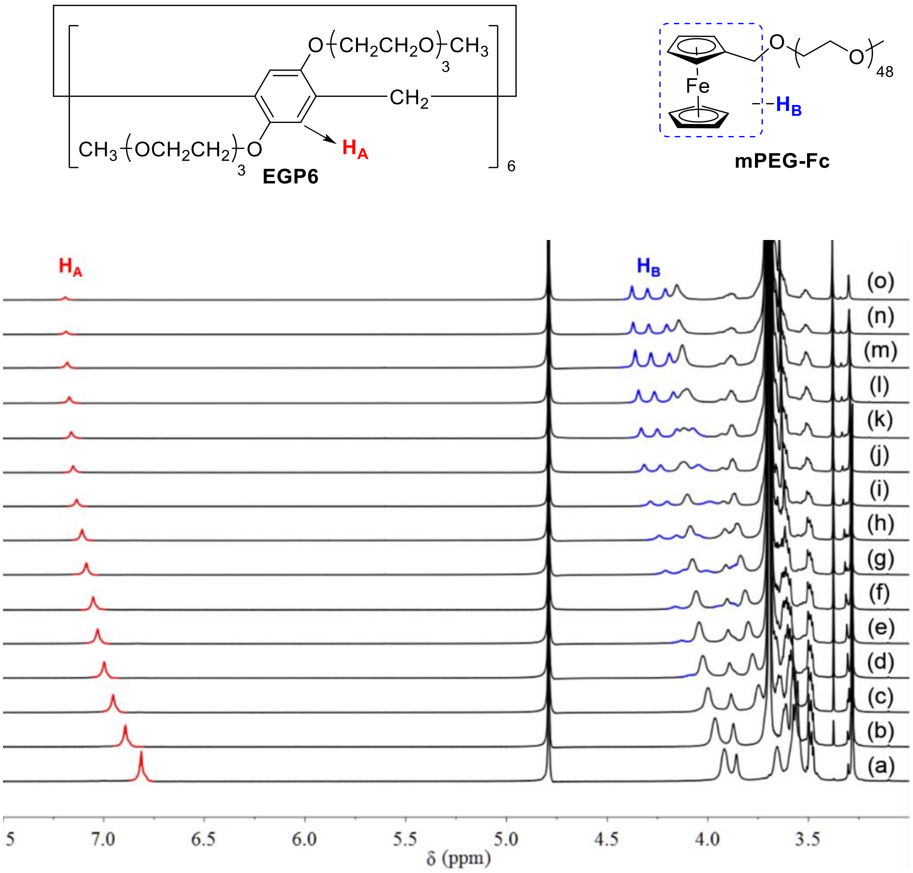

Supplementary Figure 2. Partial ^{1}H NMR spectra (400 MHz, D_2O, 298 K) of **EGP6** (2 mM) in the presence of increasing concentrations of **mPEG-Fc** (mM): (a) 0.0, (b) 1.0, (c) 2.0, (d) 3.0, (e) 4.0, (f) 5.0, (g) 7.0, (h) 9.0, (i) 13.0, (j) 17.0, (k) 20.0, (l) 24.0, (m) 30.0, (n) 36.0, (o) 40.0.

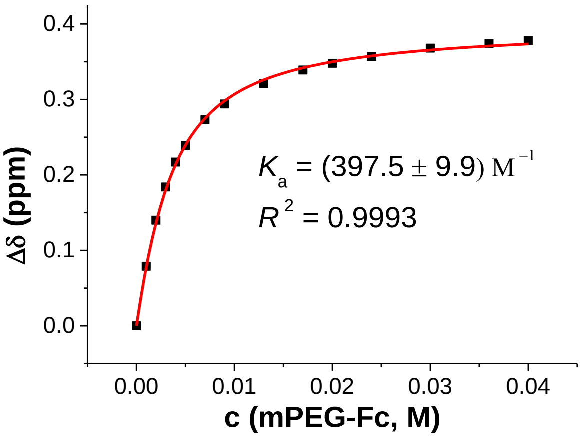

Supplementary Figure 3. Fit plot for the chemical shift changes of H_A on **EGP6** upon addition of **mPEG-Fc**.

Then, a 2D NOESY experiment was carried out to investigate the spatial conformation of such an inclusion complex, from which obvious NOE correlation signals were observed between protons H_A of **EGP6** and part of protons H_B on

ferrocene moiety of **mPEG-Fc** (Supplementary Fig. 4). However, as a control experiment, no NOE correlation signal could be observed at the corresponding chemical shift in the 2D NOESY spectrum of single **EGP6** (Supplementary Fig. 5). The above result revealed that the ferrocene moiety of the model guest **mPEG-Fc** threaded into the hydrophobic cavity of **EGP6**.

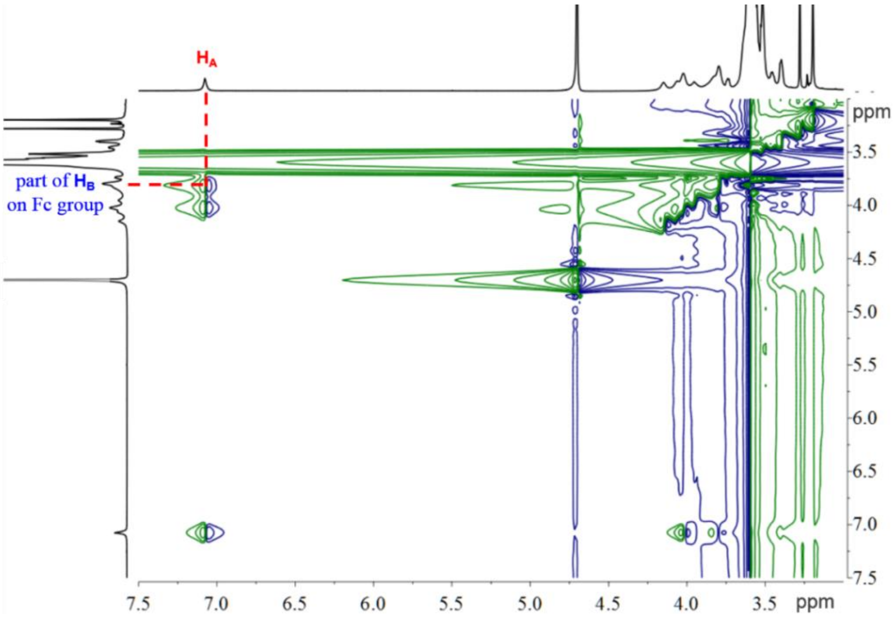

Supplementary Figure 4. Partial 2D NOESY (500 MHz, D_2O, 298 K) spectrum of **EGP6⊃mPEG-Fc**, [**EGP6**] = 5 mM, [**mPEG-Fc**] = 40 mM (part of protons H_B on ferrocene moiety of **mPEG-Fc** overlapped with the peak of **EGP6**).

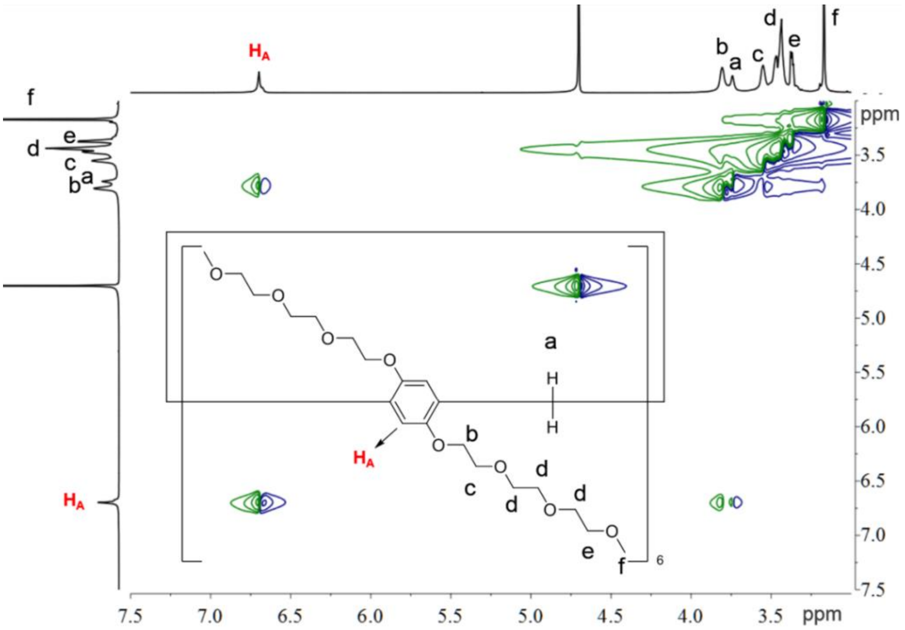

Supplementary Figure 5. Partial 2D NOESY (500 MHz, D_2O, 298 K) spectrum of **EGP6** (5 mM).

- **Swelling behavior of hydrogel after being immersed in pure water or EGP6 aqueous solution**

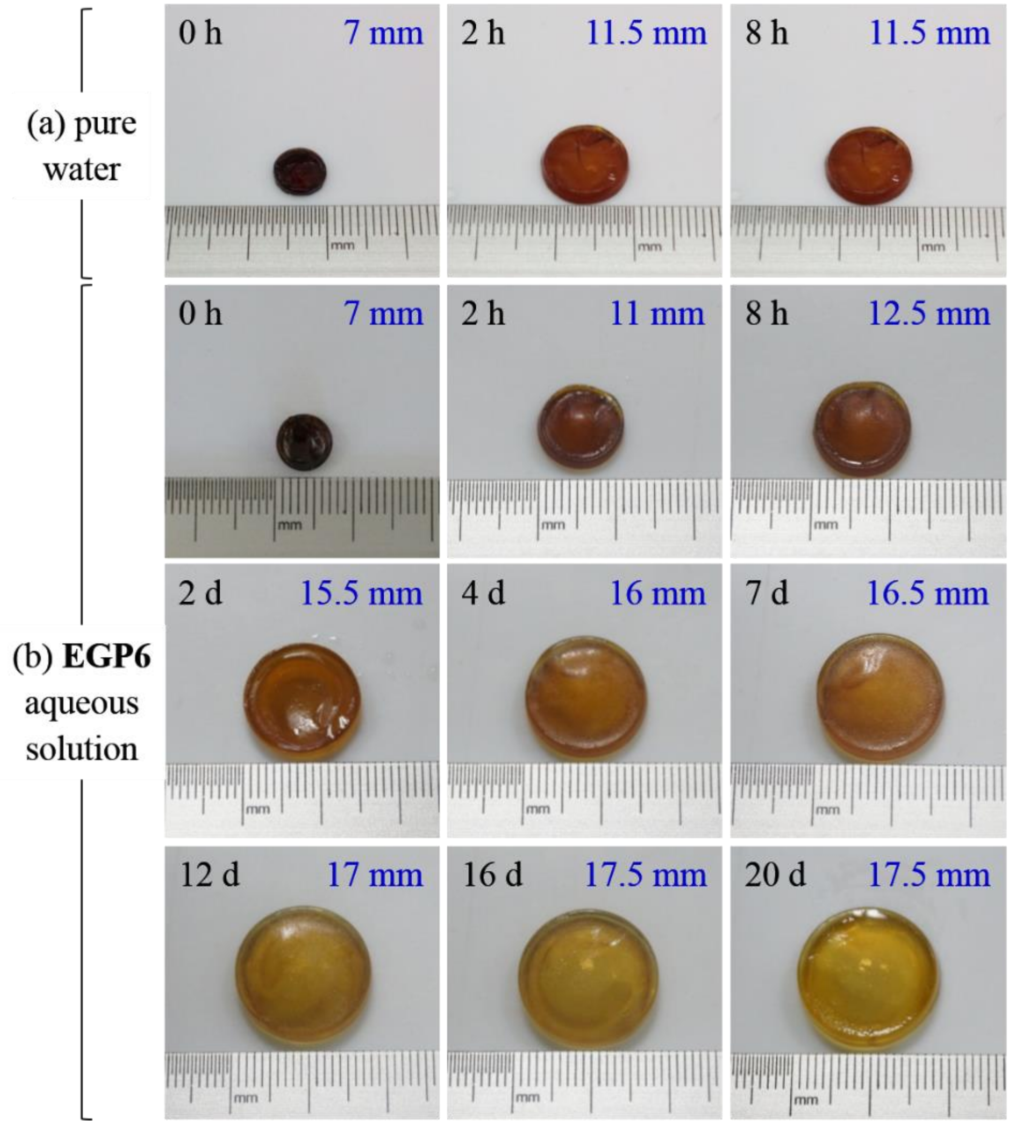

Supplementary Figure 6. Photographs of **Fc-gel** after being immersed in (a) pure water or (b) **EGP6** aqueous solution (15 mM) at 25 ℃.

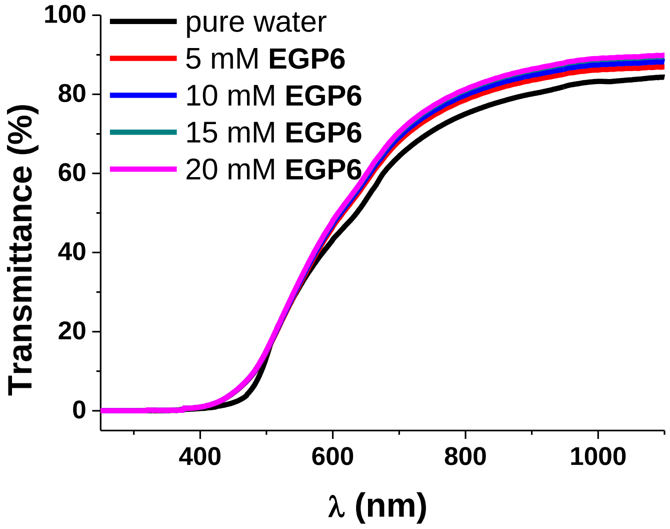

Supplementary Figure 7. Transmittances of sufficiently swollen hydrogel by immersing in pure water or **EGP6** solution of different concentrations at 25 ℃.

➢ Host–guest complexation between EGP6 and ferrocenium (Fc^+)

Due to the paramagnetic property of $\mathbf{Fc^+}$, it is difficult to study the host–guest complexation between **EGP6** and $\mathbf{Fc^+}$. In this case, the diamagnetic cobaltocenium ion ($\mathbf{Cob^+}$), which has a similar binding ability to pillararene as that of $\mathbf{Fc^+}$,[4] was selected as an analogue of $\mathbf{Fc^+}$. Consequently, the host–guest complexation between **EGP6** and cobaltocenium hexafluorophosphate ($\mathbf{Cob^+PF6^-}$) was investigated by ^{1}H NMR spectroscopy (Supplementary Fig. 8).

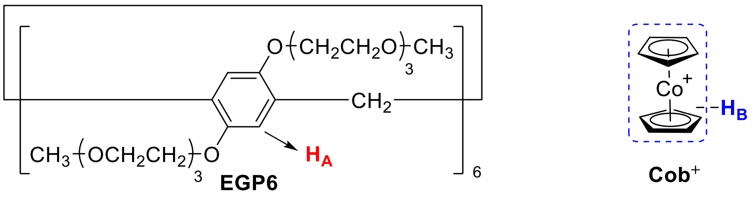

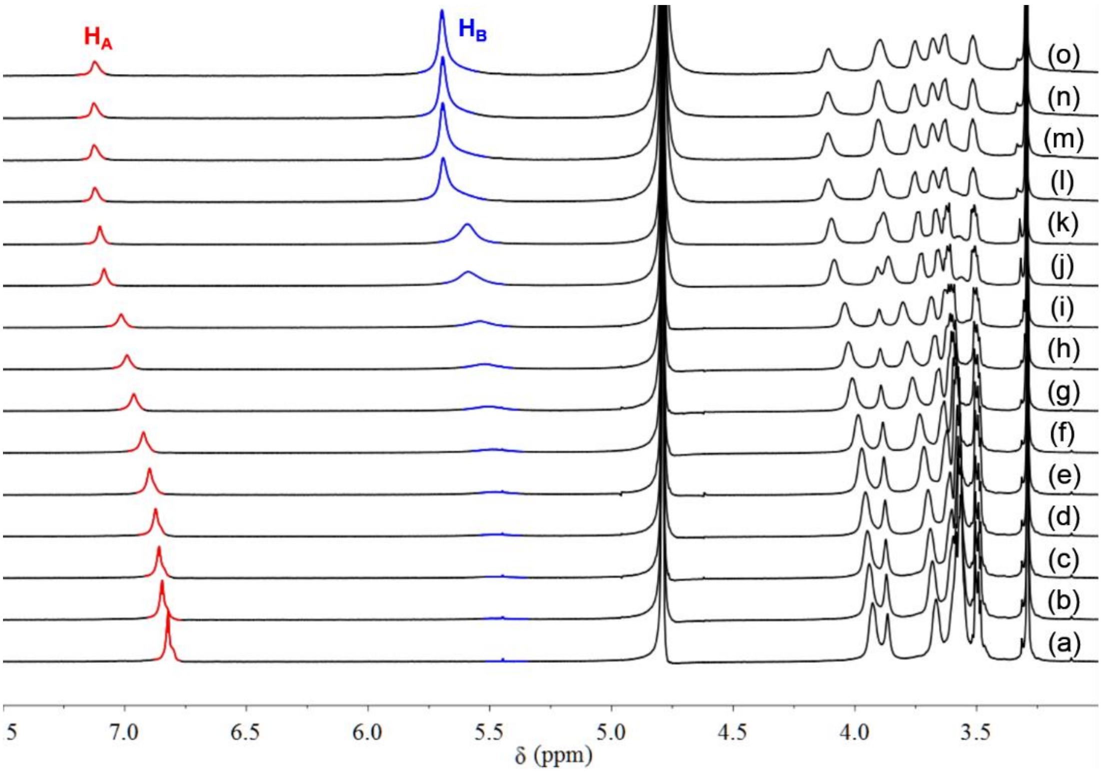

Supplementary Figure 8. Partial ^{1}H NMR spectra (400 MHz, D_2O, 298 K) of **EGP6** (2 mM) in the presence of increasing concentrations of **Cob^{+}PF6^{-}** (mM): (a) 0.0, (b) 0.5, (c) 1.0, (d) 1.5, (e) 2.0, (f) 3.0, (g) 4.0, (h) 6.0, (i) 8.0, (j) 10.0, (k) 16.0, (l) 22.0, (m) 28.0, (n) 34.0, (o) 40.0.

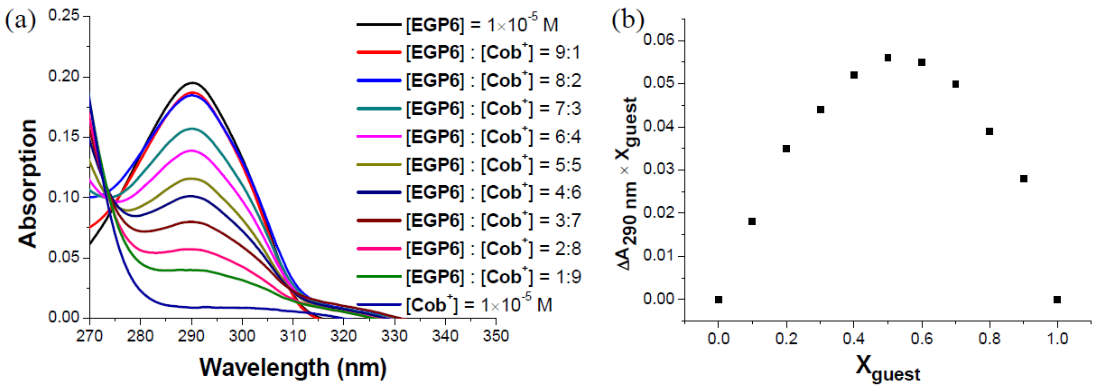

Supplementary Figure 9. (a) UV-Vis absorption of the mixture of **EGP6** and **Cob^{+}PF6^{-}** in water at different molar ratios while [**EGP6**] + [**Cob^{+}PF6^{-}**] = 1.0×10^{-5} M. (b) Job plot showing 1:1 stoichiometry of the complex between **EGP6** and **Cob^{+}PF6^{-}** by plotting the difference in absorption at 290 nm (a characteristic absorption peak of **EGP6**) against the mole fraction of guest **Cob^{+}PF6^{-}** with an invariant total concentration of 1.0×10^{-5} M in aqueous solution.

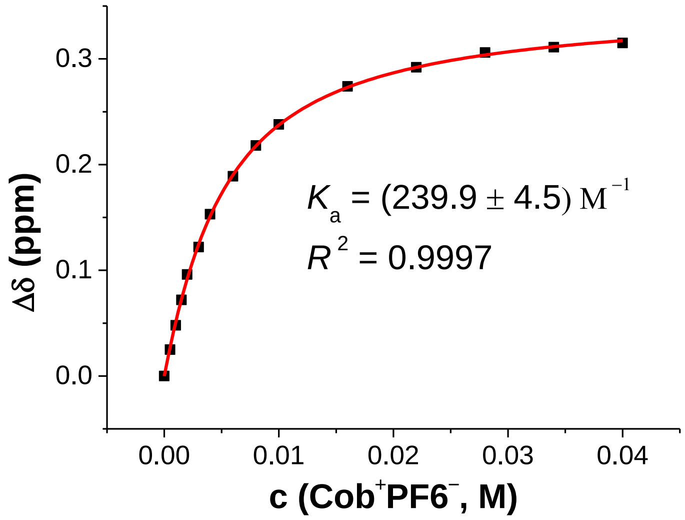

Supplementary Figure 10. Fit plot for the chemical shift changes of H_A on **EGP6** upon addition of **Cob^+PF6^-**.

➢ Variable temperature ^{1}H NMR spectra

The thermo-responsiveness of the host–guest interaction between **EGP6** and ferrocene moiety were investigated by variable-temperature ^{1}H-NMR spectroscopy (Supplementary Fig. 11). The results showed that when an aqueous solution of **EGP6** and **mPEG-Fc** was heated up to 45 ℃, the chemical shifts of ferrocene signals of the model guest **mPEG-Fc** returned to the uncomplexed state. However, the complexation between **EGP6** and **mPEG-Fc** re-formed after decreasing the solution temperature to 25 ℃. Therefore, the complexation between **EGP6** and ferrocene moiety can be reversibly controlled by heating and cooling.

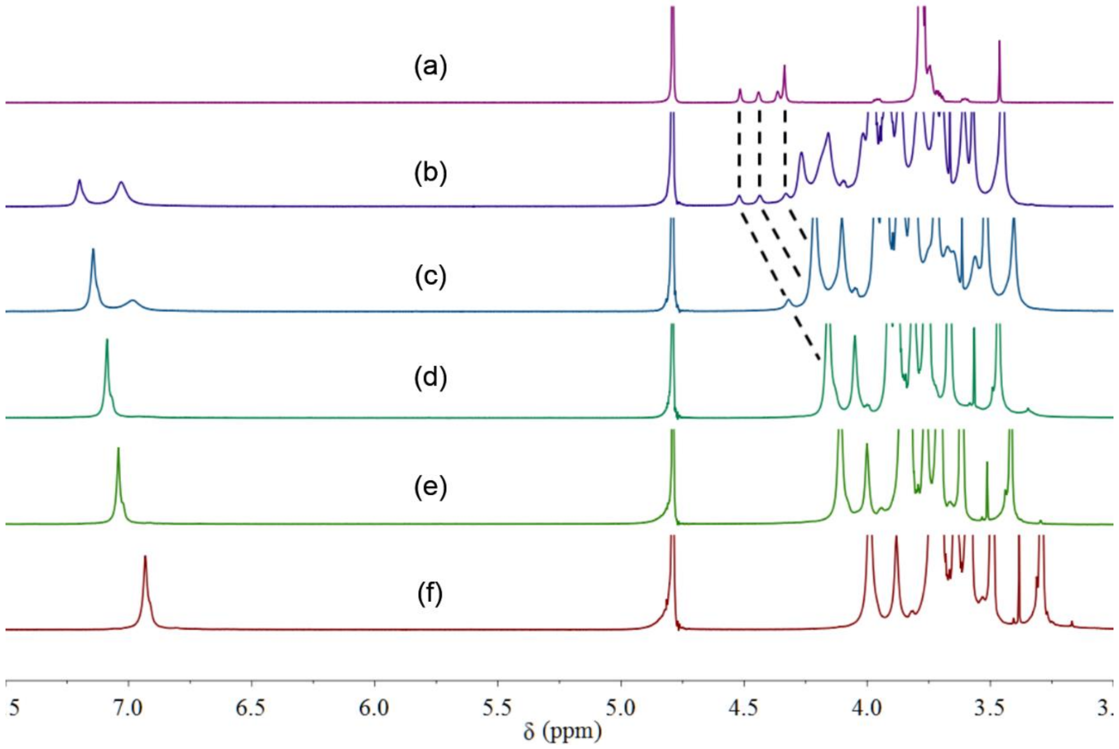

Supplementary Figure 11. Partial variable temperature ^{1}H NMR spectra (600 MHz, D_2O) of (a) **mPEG-Fc** (4 mM) at 45 ℃, and **mPEG-Fc** (4 mM) with the presence of **EGP6** (4 mM) at: (b) 45 ℃; (c) 40 ℃; (d) 35 ℃; (e) 30 ℃; (f) 25 ℃.

- **Stability of Fc-gel•EGP6 hydrogel**

The main factor determining the stability of **Fc-gel•EGP6** hydrogel is its capability to hold **EGP6** within the hydrogel without leaking out from the hydrogel backbone. To investigate the stability of **Fc-gel•EGP6** hydrogel, we alternately exposed the hydrogel to air at 25 ℃ and then to water at 40 ℃ for 50 and 100 cycles, respectively. Then the water medium was measured by UV-Vis Spectroscopy. As shown in Supplementary Fig. 12, there was almost no absorption signal at 290 nm (a characteristic absorption peak of **EGP6**), which revealed that almost no **EGP6** had leaked out from the hydrogel. So the stability of the **Fc-gel•EGP6** hydrogel is very good.

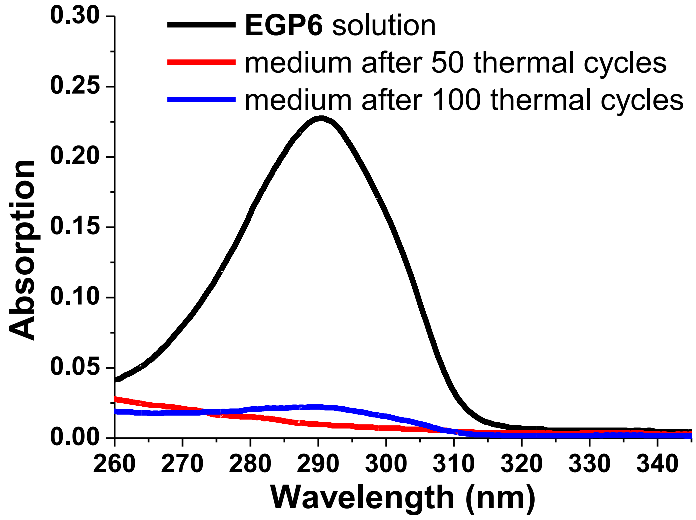

Supplementary Figure 12. UV-Vis absorption spectra of **EGP6** solution and the water medium after different thermal cycles upon alternately exposing the **Fc-gel•EGP6** hydrogel to air at 25 ℃ and then to the water medium at 40 ℃.

➢ Transmittance spectra of Fc-gel·EGP6 hydrogel

The solar modulation ability (ΔT_{sol}) and enhanced luminous transmittance (T_{lum}) of **Fc-gel•EGP6** hydrogel were calculated according to the reference method.[5] Compared with the other reported results for the mostly studied inorganic VO_2 thermochromic materials,[6] a good combination of higher T_{lum} (64.0%) and ΔT_{sol} (66.9%) was obtained for this hydrogel material.

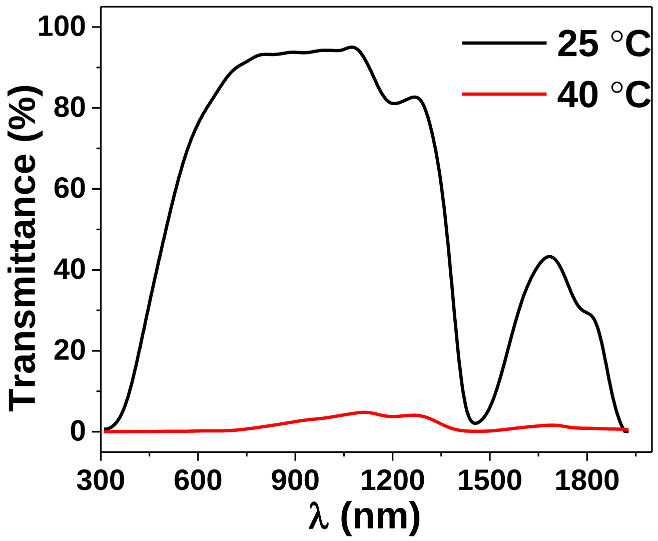

Supplementary Figure 13. Transmittance spectra of **Fc-gel·EGP6** hydrogel at 25 ℃ and 40 ℃, respectively.

Supplementary References

1. Ni, M., Zhang, N., Xia, W., Wu, X., Yao, C., Liu, X., Hu, X.-Y., Lin, C. & Wang, L. Dramatically promoted swelling of a hydrogel by pillar[6]arene–ferrocene complexation with multistimuli responsiveness. *J. Am. Chem. Soc.* **138**, 6643-6649 (2016).
2. Ogoshi, T., Kida, K. & Yamagishi, T.-a. Photoreversible switching of the lower critical solution temperature in a photoresponsive host–guest system of pillar[6]arene with triethylene oxide substituents and an azobenzene derivative. *J. Am. Chem. Soc.* **134**, 20146-20150 (2012).
3. Wang, S., Yao, C., Ni, M., Xu, Z., Cheng, M., Hu, X.-Y., Shen, Y.-Z., Lin, C., Wang, L. & Jia, D. Thermo- and oxidation-responsive supramolecular vesicles constructed from self-assembled pillar[6]arene-ferrocene based amphiphilic supramolecular diblock copolymers. *Polym. Chem.* **8**, 682-688 (2017).
4. Xia, W., Hu, X.-Y., Chen, Y., Lin, C. & Wang, L. A novel redox-responsive pillar[6]arene-based inclusion complex with a ferrocenium guest. *Chem Commun* **49**, 5085-5087 (2013).
5. Liu, C., Wang, S., Zhou, Y., Yang, H., Lu, Q., Mandler, D., Magdassi, S., Tay, C. Y., Long, Y. Index-tunable anti-reflection coatings: Maximizing solar modulation ability for vanadium dioxide-based smart thermochromic glazing. *J. Alloy. Compd.* **731**, 1197-1207 (2018).
6. Lu, Q., Liu, C., Wang, N., Magdassi, S., Mandler, D., Long, Y. Periodic micro-patterned VO_2 thermochromic films by mesh printing. *J. Mater. Chem. C* **4**, 8385-8391 (2016).
