# Supplementary material for: Warm/cool-tone switchable thermochromic material for smart windows by orthogonally integrating properties of pillar[6]arene and ferrocene
Source: Nat Commun. 2018 Apr 30;9:1737. doi: 10.1038/s41467-018-03827-3 (PMC5928112; doi:10.1038/s41467-018-03827-3)
Supplement: Supplementary file 3 — Description of Additional Supplementary Files [file 41467_2018_3827_MOESM3_ESM.pdf]

Description of Additional Supplementary Files

File Name: Supplementary Movie 1
Description: Transparency changes of Fc-gel·EGP6 hydrogel in warm color.

File Name: Supplementary Movie 2
Description: Color changes of Fc-gel·EGP6 hydrogel between warm and cool color at 25 °C.

File Name: Supplementary Movie 3
Description: Transparency changes of the oxidized Fc-gel·EGP6 hydrogel in cool color. The other formats of Supplementary Movies have also been checked according to the above guidelines.
